# When the Stones Keep Hitting, Leave No Stone Unturned: A Report of Two Cases of Adenine Phosphoribosyltransferase (APRT) Deficiency Progressing to End-Stage Renal Failure and Recurrence Post-transplant in One of Them

**DOI:** 10.7759/cureus.98883

**Published:** 2025-12-10

**Authors:** Nahush Chafekar, Babaniji Omosule, Murad Aldujaili, Manivarma Kamalanathan

**Affiliations:** 1 Renal Medicine, Royal Wolverhampton Hospital NHS Trust, Wolverhampton, GBR; 2 Renal Medicine, University Hospitals Birmingham NHS Foundation Trust, Birmingham, GBR; 3 Nephrology, Royal Wolverhampton Hospital NHS Trust, Wolverhampton, GBR

**Keywords:** adenine phosphoribosyltransferase (aprt) deficiency, graft loss, nephrolithiasis, recurrence post transplant, renal failure

## Abstract

Adenine phosphoribosyltransferase (APRT) deficiency is a rare autosomal recessive disorder of the purine pathway that results in excessive production of 2,8-dihydroxyadenine and subsequent nephrolithiasis and crystal nephropathy. It has varying presentations, and this, in combination with its rarity, often leads to delayed diagnosis. It is also associated with recurrence in the allograft post transplant with potential for graft loss, especially if the diagnosis was not made.

We present two cases of APRT deficiency in two men with varying presentations and progression to end-stage renal failure. There was a recurrence post renal transplant in one of them who had delayed graft function, and the diagnosis was made at this point. Early diagnosis and prompt initiation of therapy are critical in preventing the progression of disease.

## Introduction

Adenine phosphoribosyltransferase (APRT) deficiency is an autosomal recessive disorder of purine metabolism that is characterized by the development of radiolucent stones in the kidney and urinary tract and resultant progressive chronic kidney disease (CKD) secondary to crystal nephropathy [1,2]. It is rare, with an estimated prevalence of 1:27,000 among the Japanese and 1:50,000-1:100,000 among whites [2]. It is often an under-recognized cause of recurrent kidney stones and, if left untreated, progresses to end-stage renal disease with reported recurrence and graft loss post-transplant [3,4].

APRT deficiency is caused by mutation of the APRT gene on chromosome 16, and APRT is utilized in purine synthesis in the formation of adenosine monophosphate and inorganic pyrophosphate from adenine and 5-phosphoribosyl-1-pyrophosphate [5]. APRT deficiency thus hinders the recycling of adenine (a DNA building block) and thus cellular energy storage, by leading to its conversion by xanthine dehydrogenase (XDH) to 2,8-dihydroxyadenine (2,8-DHA), a poorly soluble substance that then causes massive crystalluria, stone formation, and crystalline nephropathy [3,5,6].

We report two cases of 2,8-DHA crystalline nephropathy caused by APRT deficiency in a 19-year-old male who initially presented with acute kidney injury and obstruction, and a 53-year-old male with unexplained progressive CKD, and the diagnosis was only made post kidney transplant following delayed graft function.

## Case presentation

Case summary one

A 19-year-old male initially presented with several episodes of diarrhea and vomiting. He had a deranged renal function with a creatinine of 208 µmol/l (reference range: 64-111 µmol/l; previously known baseline was 116 µmol/l). Initial diagnosis of pre-renal acute kidney injury (AKI) secondary to viral gastroenteritis was made, and he was rehydrated with intravenous fluids, which led to a marginal improvement in his renal function to 176 µmol/l. He was discharged from the hospital following resolution of symptoms and planned for subsequent follow-up in the renal clinic.

His ultrasound scan at that time showed normally sized kidneys with increased cortical echogenicity and reduced cortico-medullary differentiation bilaterally, which suggested there was an underlying chronic kidney disease. In addition, the right kidney was mildly hydronephrotic, and there was a 17 mm hyperechoic focus in the right side of the urinary bladder, raising the possibility of stones at the vesico-ureteric junction. His urine dip was negative for blood and protein, and he was referred to the Urologist, who offered him an outpatient appointment as renal function appeared to be improving.

On follow-up appointment, four weeks after his discharge, it was noted that his renal function had deteriorated slightly with an increase in serum creatinine to 201µmol/l, but he had a normal immunology screen. Two weeks later, he was readmitted to the hospital with right flank pain, raised inflammatory markers, and his serum creatinine had further increased to 328 µmol/l. Repeat ultrasound scan at the time showed his right kidney was now moderately hydronephrotic, but there was still no clear evidence of an obstructing calculus. CT KUB, however, revealed an 8mm obstructive calculus in the proximal right ureter with hydroureteronephrosis, and he then had a right retrograde ureteric stent inserted. The left kidney was unobstructed. He was discharged with a plan for future right ureteroscopy +/- laser treatment for ureteric stones.

Three months post stent insertion, he was admitted to a local hospital with worsening loin pain and deteriorating kidney function while on holiday abroad. CT scan showed blockage of his right ureteric stent and he had a right nephrostomy inserted along with a course of antibiotics. He returned to the UK and was readmitted due to worsening symptoms of right flank pain and malaise. Repeat CT scan showed a patent right side nephrostomy tube and there was no evidence of any visible calculus. His renal function was significantly more impaired, with his serum creatinine peaking at 600 µmol/l. 

**Figure 1:**
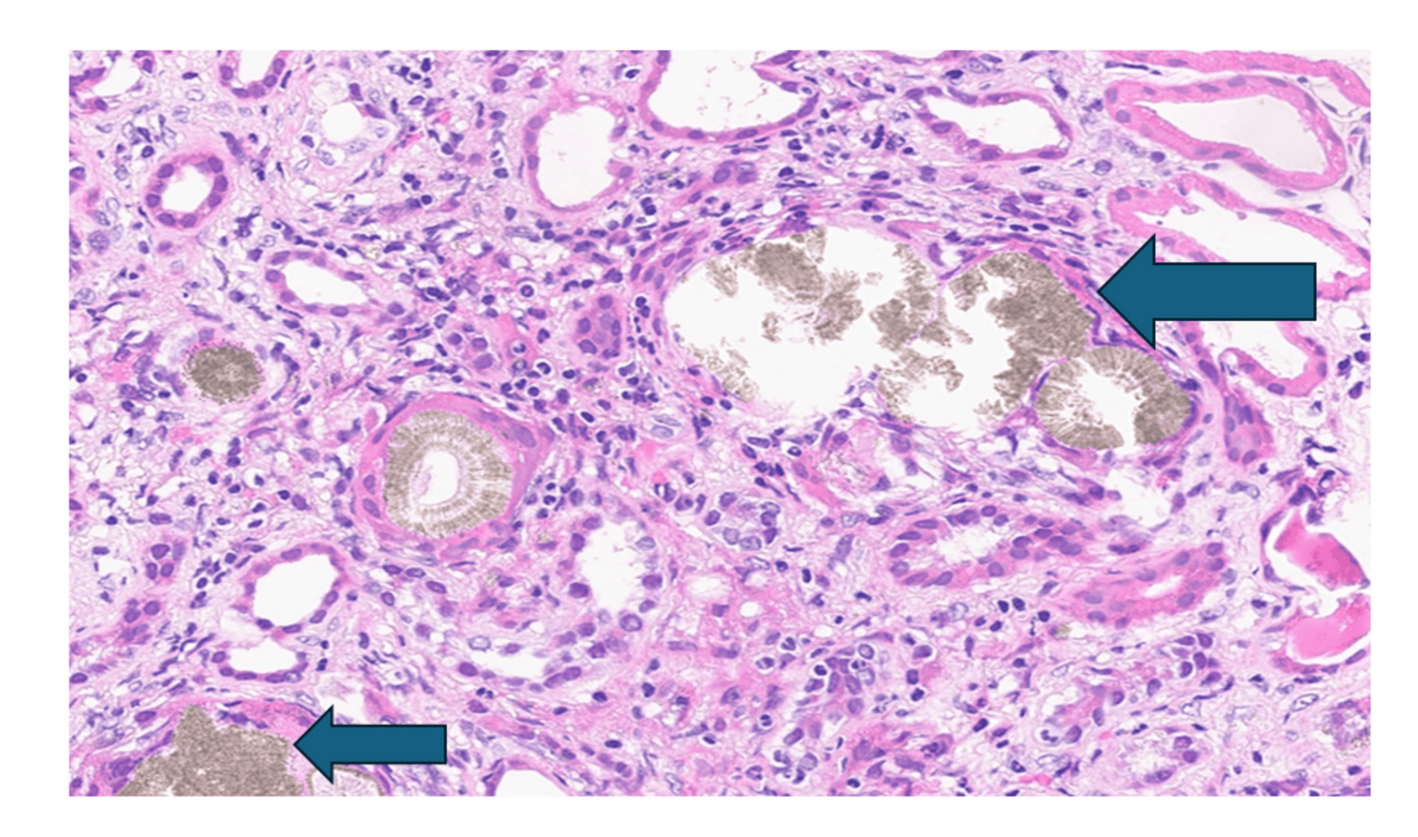
H & E stain with blue arrows showing crystals in tubules

**Figure 2:**
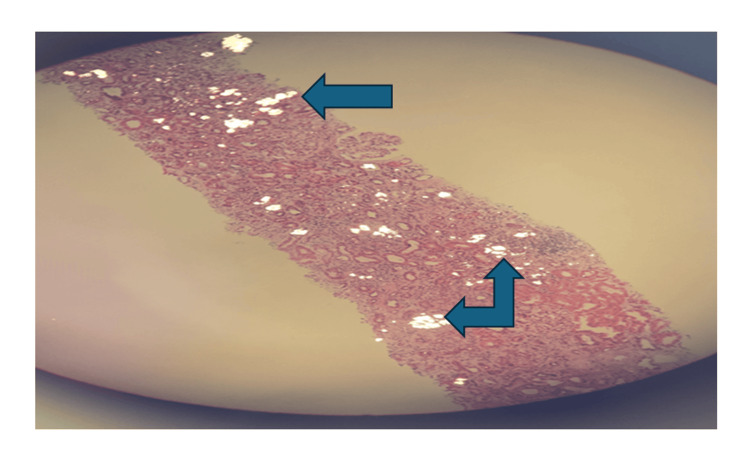
Arrows showing crystals under polarized light displaying bright birefringence

The presence of significant renal dysfunction, absence of proteinuria, and unobstructed kidneys led to a decision to perform a kidney biopsy due to suspicion of an intrinsic kidney disease. This subsequently showed features of obstructive intraluminal crystalline casts with unusual bright green color in many tubules (Figure 1), moderate acute tubular injury, and mild interstitial neutrophil infiltration suggestive of acute pyelonephritis. These crystals also displayed bright birefringence under polarized light (Figure 2). After discussion in our renal histopathology MDT meeting, the possibility of crystallite/tubular nephropathy was considered, and he was commenced on a short course of steroids. Twenty-four-hour urine oxalate excretion was normal and following discussion with the local tertiary specialist center, an APRT deficiency screen was sent. He was then commenced on empirical Allopurinol and Pyridoxine.

His renal function continued to deteriorate, and he subsequently commenced hemodialysis. Genetic testing confirmed he had homozygous APRT deficiency with APRT in red blood cells of 2nmol/Hr/mgHb (reference range 14-39). 2,8-DHA was also detected in his plasma. No positive family history was present and hence genetic counselling was advised.

Case summary two

A 53-year-old man, known CKD 3, was initially referred to the renal clinic on account of derangement in renal function with an increase in creatinine to 400 from 198 µmol/l. Two years prior to this, his creatinine was 118-141µmol/l. He had a previous history of renal stones and chronic hydronephrosis of the right kidney but he did not have any procedure for this or stone analysis. He also had a family history of renal stones and is a known hypertensive. His CT scan showed a small right kidney with no identifiable stones in the kidney or ureter. Isotope Renogram also showed a very slow uptake within the right kidney with a split renal function of 85% in the left kidney and 15% in the right kidney. He then had a biopsy of his left kidney, which showed chronic interstitial damage with calcium oxalate crystal deposition. He had an elevated parathyroid hormone (354ng/L; reference range: 10-65ng/l), but he was normocalcemic with a calcium of 2.38 mmol/l (reference range: 2.20-2.60 mmol/l). He had hypocalciuria and a moderately elevated urinary oxalate excretion of 0.52mmol/24 hours. His renal function continued to deteriorate, and he subsequently commenced hemodialysis. He eventually had an altruistic live donor renal transplant. 

He unfortunately had delayed graft function and multiple transplant biopsies then revealed positive birefringent crystals, with each biopsy showing a progressive increase in oxalate deposition. A possibility of primary hyperoxaluria as the etiology of his renal failure was strongly entertained at this point, and he was commenced on high-dose pyridoxine. He however had normal plasma and urine oxalate and the genotype screen for primary hyperoxaluria was negative. Urine crystal analysis revealed typical maltese cross crystals and APRT deficiency was subsequently confirmed through red cell and enzymatic analysis. He was commenced on Allopurinol and Feboxustat and eventually managed to hold off dialysis after 17 months of dialysis post-transplant. His estimated glomerular filtration rate at this time was between 12 and 14mls/min.

Three years post transplant, he was admitted for elective parathyroidectomy, which was complicated by type 2 respiratory failure secondary to aspiration pneumonia, and a myocardial infarction requiring urgent inpatient coronary artery bypass graft and dialysis. He remained dialysis dependent after this and sadly died after a few months.

## Discussion

While this enzyme deficiency is already present at birth, symptoms of APRT deficiency may manifest anytime from early childhood to the seventh-eighth decade of life, but delayed diagnosis made late in the course of the disease is often the norm [3,5]. The two patients presented in the second and sixth decades, respectively, but with varied presentations. The commonest clinical manifestation of APRT deficiency is urolithiasis, and this is the first presentation in 54-73% of cases [3,7]. Other clinical features include episodes of AKI, haematuria, lower urinary tract symptoms, and progressive CKD requiring renal replacement therapy [7]. While the first patient initially presented with AKI in the second decade of life, he then had subsequent presentations attributable to obstructive stone disease and progression of kidney disease. The second patient presented in the sixth decade with unexplained progressive CKD, but the diagnosis was only made following delayed graft function post-transplant. The second patient also gave a history of previous nephrolithiasis. Late diagnosis was central to both patients, and this is often so due to the rarity and low suspicion of disease. As such, about 70% of cases are diagnosed late, and this has implications for potential treatment and retarding the progression of the disease [7]. A systematic review reported that APRT deficiency was diagnosed in 83.3% of patients post transplant, and this has far-reaching implications for graft survival as seen in our second patient. They also reported that the average graft survival time was 19.5 months, while only a third of patients had stable graft function on follow-up [7].

Diagnostic modalities include stone analysis, isolating 2,8-DHA crystals in urine or renal biopsy, genetic analysis, or measurement of APRT activity in erythrocytes [5,8]. However, making the diagnosis of APRT deficiency is also extremely variable, just like the clinical course of the disease, and this is attributable to the radiolucent composition of DHA crystals, which often leads to a misdiagnosis of uric acid stones and the fact that these crystals also mimic oxalate crystals [1]. Various characteristics of these crystals have been described, ranging from birefringence to multiple brownish-green or brown stains with H&E and PAS, light blue stains with Trichome, and black stains with Jones Methenamine Silver. The shape has also been described as needle-, rod- or rhomboid-shaped and as annular formations of striated crystals, or as fan-like or irregular clusters. Typical 2,8-DHA crystals are seen in urine with a characteristic central maltese cross pattern, but the characteristic features of 2,8-DHA crystals are often not present on renal biopsy [1,5]. The first patient’s renal biopsy was suggestive of crystal nephropathy, although the exact crystal was not identified. However, APRT activity in erythrocytes was measured and found to be low. Figure 1 shows these brownish-greenish crystals in the tubules of the patient, while Figure 2 shows birefringence under polarized light. The 2,8-DHA crystal precipitation, which causes severe tubulointerstitial injury, can be seen on biopsy in a few instances [5]. While the renal biopsy in our patients did not identify these definite 2,8-DHA crystals, they might have been responsible for the tubulointerstitial injury seen in both patients, but the second patient had the typical Maltese crystals in his urine.

There is a role for the timely initiation of xanthine dehydrogenase inhibitors in retarding the progression of disease; however, this is often hindered by late diagnosis of the disease [3,8]. While the index patient was commenced on allopurinol late in the course of the disease with no resultant benefit, the second patient was commenced on both allopurinol and feboxustat shortly after his transplant, and he had partial renal recovery after 17 months on dialysis.

## Conclusions

In conclusion, due to the rarity of the disease and the huge variability in presentation, delay in diagnosis, as was the case in both patients, is still often the norm. A high index of suspicion is required to diagnose APRT deficiency, and there should be a low threshold to consider this possibility, especially in patients presenting with urinary stones and renal impairment, early-onset urinary stones, frequent recurrence of stones, and unexplained renal failure. Early diagnosis and prompt initiation of therapy are key in retarding the progression of the disease.
